# Effects of the Swedish physical activity on prescription model on health-related quality of life in overweight older adults: a randomised controlled trial

**DOI:** 10.1186/s12889-015-2036-3

**Published:** 2015-07-21

**Authors:** Sven JG Olsson, Mats Börjesson, Elin Ekblom-Bak, Erik Hemmingsson, Mai-Lis Hellénius, Lena V Kallings

**Affiliations:** Department of Sport and Health Sciences, Swedish School of Sport and Health Sciences Stockholm (GIH), Box 5626, 114 86 Stockholm, Sweden; Department of Cardiology, Karolinska University Hospital, Stockholm, Sweden; Department of Medicine, Huddinge, Karolinska Institutet, Karolinska University Hospital, Stockholm, Sweden; Department of Medicine, Solna, Karolinska Institutet, Stockholm, Sweden

**Keywords:** Clinical research, Health promotion, Intervention study, Older adults, Prescribed physical activity, Health related quality of life, Overweight

## Abstract

**Background:**

The effects of physical activity on prescription (PAP) on health-related quality of life (HRQoL) in overweight adults are unclear. We therefore aimed to explore the effects of the Swedish PAP model on HRQoL in overweight older adults.

**Methods:**

Participants were recruited from a cohort of men and women born between 1937 and 1938, and living in Stockholm County. Inclusion criteria were; insufficiently physically active, i.e. <30 min of at least moderate intensity physical activity (PA) per day; body mass index >25 kg/m^2^; and waist circumference ≥102 cm (men) or ≥88 cm (women). Altogether, 101 individuals, aged 67 years, were randomly assigned to two parallel groups: intervention group (*n* = 47) receiving individualised PAP or control group (*n* = 54). The 36-item Short Form Health Survey (SF-36) was administered before and after the six months intervention. Main outcomes were the SF-36 physical component summary (PCS) and mental component summary (MCS) scores. Intention to treat analysis was utilised. Regression analysis was performed to assess whether changes in PA and body weight affected changes in HRQoL.

**Results:**

At the six months follow-up, regarding the MCS score, the intervention group had improved significantly more (median: 4.4 [interquartile range (IQR): −2.4 to 23.3]) vs (median: 0.0 [IQR: −4.0 to 4.9]); *p* < 0.05) and a higher proportion of participants had attained relevant improvements (OR 2.43 (95 % CI 1.00–5.88) *p* < 0.05) compared to the controls. A within group improvement in the PCS score (median: 3.8 [IQR: −1.9 to 19.5] *p* < 0.05) was found in the intervention group. Changes in PA and body weight had a small, but significant, mediating effect on the changes in HRQoL.

**Conclusions:**

PAP had a positive effect on HRQoL, measured by the SF-36 MCS, but no significant between group effect was seen on the PCS in overweight older adults. These effects were, to some extent, mediated by changes in PA and body weight. Our findings support clinical use of the Swedish PAP model.

**Trial registration:**

ClinicalTrials.gov NCT02320760.

## Background

Physical activity (PA) is beneficial for promotes health across all age categories. However, the level of PA decreases with age, particularly after age 65 [1]. The prevalence of lifestyle diseases also increase with age. This causes subsequent decreases in health related quality of life (HRQoL), an important measure of burden of disease. The Centers for Disease Control and Prevention describe HRQoL as “*a broad multidimensional concept that usually includes self-reported measures of physical and mental health*” [2]. Due to increased mean life expectancy we need to ascertain HRQoL throughout the extended older adulthood. Both positive effects [3–5], and limited evidence or absence of effects [6–11], of PA on HRQoL in older adults are reported from randomised controlled trials (RCT). However, heterogeneity in methods and measured HRQoL domains complicate the interpretation [10, 12].

Different models for prescribed PA exist internationally and have shown varying degree of efficacy. A difficulty lies in reviewing the effect of different models because they are of different constructs, provided to different populations or patient cohorts in different sociocultural environments. Recent systematic reviews unanimously concluded that there is insufficient evidence to recommend Exercise Referral Schemes (ERS) over advice or counselling interventions [13–15]. Nevertheless, further scientific evaluation is called for in these reviews. There are different prescribed PA models from Denmark [16], the Netherlands [17], and Finland [18] and they all, just like the ERS’, vary in construct to some extent. The Swedish physical activity on prescription (PAP) model has five components: (a) patient-centred counselling, (b) written prescription of individualised PA using (c) the text book *FYSS* (*Physical Activity in the Prevention and Treatment of Disease*) for evidence-based prescriptions [19], (d) follow-up assessments, and (e) collaboration with societal organisations [20]. A good summary of the model is given by Raustorp and Sundberg [21].

The Swedish PAP model has been shown to increase PA and improve HRQoL in a heterogeneous patient cohort [22], and in hypertensive patients [23] and to reduce cardiovascular risk factors without decreasing HRQoL in healthy men aged 35–60 years [24]. However, further scientific evaluation of effects on HRQoL of the Swedish PAP model is needed as the literature is scarce [25]. Additionally, evaluation of the effects on health of different models for prescribing PA is needed as the international literature is inconclusive [13–18].

The study at hand reports secondary outcomes from a RCT of which the primary aim was to assess the effect of the Swedish PAP on the level of PA in overweight older adults with abdominal obesity. PA and body weight improved significantly in favour of the participants who received PAP. These results are reported in detail elsewhere [26, 27]. It was hypothesised, a priori, that the Swedish PAP could have a direct effect on HRQoL, and a posteriori, that such an effect could be mediated by changes in PA and body weight. Further, to our knowledge, no RCT to examine the effect of the Swedish PAP model on HRQoL has been published. The objective of this study was therefore to examine the effect of the Swedish PAP model, and the influences of changes in PA and body weight, on HRQoL in overweight older adults.

## Methods

### Participants

Participants were recruited from a cohort of Stockholm County citizens born between 1937 and 1938 who had taken part in a health screening survey (*n* = 4232) [28]. Inclusion criteria were: healthy but insufficiently physically active (<30 min of at least moderate intensity PA per day), a body mass index (BMI) between 25 and 40 kg/m^2^ and waist circumference ≥102 cm (men) or ≥88 cm (women). Exclusion criteria included self-reported pharmacological treatment for hypertension, hyperlipidaemia, type 2-diabetes, or serious chronic disease. The proportion of older people (>65 years) in the population is increasing worldwide. Because most chronic diseases manifest later in life and PA decreases with age, it is urgent to develop and evaluate methods for promoting PA and HRQoL in the elderly. The rationale for the inclusion criteria was therefore to reach an older (>65 years) group with insufficient levels of PA, overweight and abdominal obesity.

**Fig. 1:**
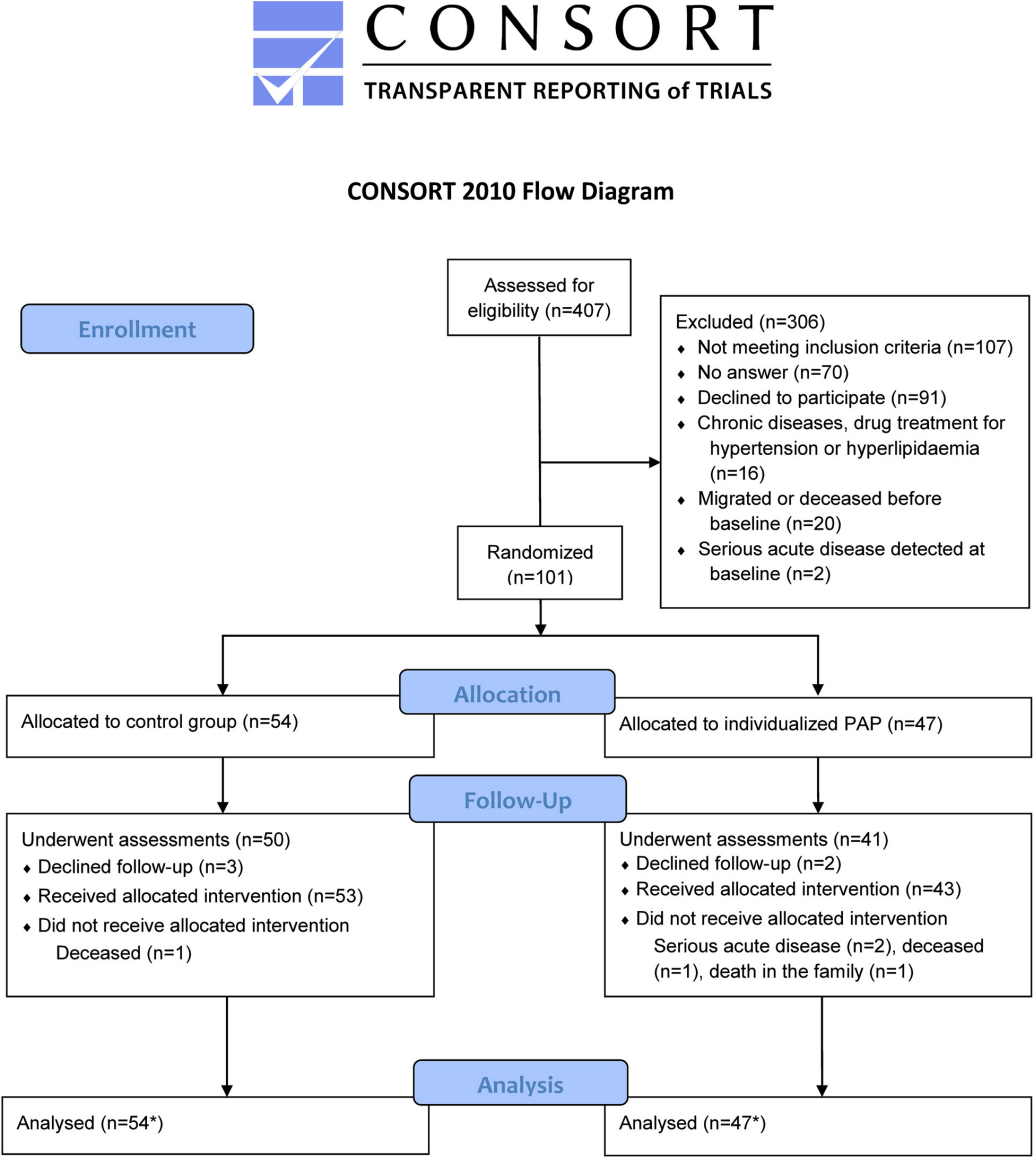
Flowchart of all participants from the screening to the six month follow-up. *Note:* PAP = Physical activity on prescription. *Intention to treat analysis with last observation (baseline) carried forward for participants that did not provide follow-up data (*n* = 2), and single missing data points at follow-up (each SF-36 item counting as data point)

In 2005, an invitation and a pre-screening questionnaire were sent to 407 individuals who had met the inclusion criteria during the initial screening in the late 1990s. Out of the 246 participants who agreed to take part in the present study, 116 met the inclusion criteria after the screening process. Fifteen of these participants were excluded before randomisation due to migration, death, or serious acute illness (Fig. 1).

### Design

The six months blinded RCT was conducted at the Obesity Unit of the Karolinska University Hospital in Huddinge, Sweden. The intervention group received treatment according to the Swedish PAP model [20, 27], and the control group received general written advice on PA and health. Randomisation sequence was superimposed on the calendar days of the baseline assessments to prevent social interaction between participants in the two groups. The staff delivering the intervention could not be blinded to group allocation. Measurements were performed just before (baseline) and right after the six months intervention (follow-up), and included pedometry, anthropometrics, a venous blood sample, blood pressure, PA diary [26, 27], and a questionnaire (hereby referred to as the questionnaire). The questionnaire contained items regarding demographics, lifestyle, health, PA, food habits, morbidities, and problems that inhibit everyday tasks, social interaction, and sex life. The SF-36 and the item regarding sitting time from the last 7-day short form International Physical Activity Questionnaire (IPAQ) were also included in the questionnaire.

### The intervention

For ethical reasons, the control group received one page of general written information about the importance of PA for health. The control group were not supported in any other way regarding PA behaviour during the course of the trial. The intervention group received treatment according to the Swedish PAP model [26, 27] in addition to the same written information the control group received. Directly after the baseline assessments, a physician briefly raised the question of increased PA for health with each participant. This was followed by 30 min of individualised patient-centred counselling, resulting in a prescription for PA by a health-care professional trained in motivational counselling for PA, The participants received one counselling session each during the trial. The PAP counselling was individually tailored and encompassed an agreement for increasing PA including individual goals, which were summarised and written on a prescription form. Each individualised written prescription included the specified modality of PA, intensity, frequency and duration of the different activities, and the rational for the PAP. All participants were encouraged to gradually increase their PA level to meet the recommended level of PA, i.e. accumulate 30 min or more of at least moderate intensity PA on most, preferably all, days of the week and include both aerobic and strength training, as well as exercise for improved flexibility and balance [29]. They were also encouraged to decrease their time spent being sedentary. Within one month of the baseline assessments, a 60 min group session was held with the intervention group. It included a lecture by a physician regarding PA and health and time for discussion and questions. Each person in the intervention group received a letter from the physician, with individualised advice on increase of PA, based on the baseline assessments. All intervention group participants knew that there would be a follow-up PAP counselling session after the six months assessments. The theoretical framework for the intervention was based on social cognitive theory, the transtheoretical model, motivational interviewing and supportive environment [30–33]. The counselling provided was adjusted to each participant’s readiness to change.

### Quality of life

The 36-item Short Form Health Survey (SF-36) (IQOLA SF-36 Standard Swedish Version 1.0) was part of the questionnaire. It consists of 36 items used to score eight scales: limitations in physical activities because of health problems (physical functioning), limitations in usual role activities because of physical health problems (role physical), bodily pain, general health perceptions (general health), energy and fatigue (vitality), limitations in social activities because of physical or emotional problems (social functioning), limitations in usual role activities because of emotional problems (role emotional), and psychological distress and well-being (mental health). Two summary measures, the physical component summary (PCS) and the mental component summary (MCS), are derived from the first and last four scales, respectively [34–38]. The SF-36 is the most extensively validated and used generic tool for measuring HRQoL, including among older adults [12, 39, 40]. The SF-36 data were handled and scored following previously published instructions [38]. All scores range from 0 to 100, representing the worst and best possible score. The minimum value for a clinically important improvement in the >SF-36 scores was identified as a baseline value <88 and follow-up value ≥88 (hereby referred to as “change to top-score”). This method identifies those that respond with the maximum score on the majority, or all, of the ordinal scales corresponding to a certain SF-36 score. It is possible to score less than maximum on half, or more, of the ordinal scales and still achieve a SF-36 score larger than 88 regarding bodily pain, vitality, and mental health; therefore, in these cases, the change to top-score method is not, theoretically, valid. The change to top-score method is inspired by the work of Velanovich [41], and Spratt [42]. Velanovich argued that becauseSF-36 data are very often not normally distributed, and left skewed, and the SF-36 scores are derived from ordinal scales ranging from two to six grades, appropriate statistical techniques should be used. The traditional normalisation of the SF-36 data, i.e. converting the ordinal scales to a scale of 1 – 100, does not guarantee a normal distribution. Therefore, in cases when the mode and the median are both at maximum score, i.e. 100, Velanovich has suggested using the difference in proportions of individuals scoring 100 to enable group comparisons [41]. However, Velanovich did not suggest any methods for evaluating individual changes in SF-36 scores. According to Spratt a minimal clinically important difference (MCID) must be larger than the standard error of measurement and have clinical relevance. Regarding the SF-36 score for physical functioning, Spratt found the standard error of measurement ranged from 5 to 20 at six month follow-up, and a cut-off of 85 to be useful for determining a clinically important improvement [42]. Bjorner et al. have found that a ≥10 point decrease in SF-36 vitality score was significantly associated with increased odds of negative outcomes such as hospitalisation, and mortality. However, changes in SF-36 were not compared to positive health outcomes [43], and the ≥10 point decrease cut-off lies within the range of SF-36 standard error of measurement found by Spratt [37, 42]. Further, the standard error of measurement ranged from 12 to 17 in Swedish normative population data for men and women 65 – 69 years old [37, 38]. Therefore, to enable analysis of clinically significant changes in the SF-36 scores bodily pain, vitality, and mental health, we used a >20 point change in SF-36 scores as a marker of a MCID in individuals from baseline to follow-up (hereby referred to as ‘>20 point improvement’).

### Physical activity and sedentary behaviour measures

The Yamax Digiwalker SW-200 pedometer (Yamax Corporation, Tokyo, Japan) [44, 45] was used to measure daily steps and the participants were instructed to wear it at all times for seven consecutive days, except when sleeping, bathing, or showering. The participants were instructed to register daily steps in a pre-printed seven day PA diary at baseline and follow-up assessments. In this diary they also registered frequency and duration of all physical activities, which were self-rated according to Borg’s Rate of Perceived Exertion (RPE) scale, ranging from 6 (no exertion at all) to 20 (maximal exertion imaginable) [46]. Oral instructions on how to use the diary and Borg’s RPE scale [47] were given individually to all participants, who also practiced, under supervision, using the Borg scale while cycling on an ergometer. The intensity of PA was later categorised as “light” (RPE 10–11), “moderate” (RPE 12–13), or “vigorous” (RPE ≥14) [48]. At baseline and follow-up assessments all participants were asked to complete the questionnaire. Time per day spent sitting was assessed with an item from the last 7-day short form IPAQ, which was part of the questionnaire [49]. A validated question for leisure time PA during the last 12 months was also part of the questionnaire, and included four categories ranging from sedentary leisure time (<2 h of light PA/week) to regular exercise (≥3 times/week, ≥30 min each time) [50].

### Statistical analysis

All statistical calculations were performed using STATISTICA 11 (StatSoft, Inc., Tulsa, OK, USA). An alpha level of 0.05 was used to determine statistical significance. Normality was assumed if a Lilliefors corrected Kolmogorov-Smirnov test and a Shapiro-Wilk test generated non-significant results, z-scores of skewness and kurtosis were within zero +/− 1.96 standard error of the respective statistics. An intention to treat approach, where missing data (i.e. single missing data points at follow-up or when a participant had not taken part of the follow-up assessments at all (*n* = 2)) was replaced by last observation carried forward, was used. A Wilcoxon matched pairs test was used to analyse differences in the SF-36 scores between baseline and follow-up within each group. Mann–Whitney U Tests were used to analyse differences between the intervention and control group in the SF-36 scores at baseline, and in changes in the SF-36 scores from baseline to follow-up. Nonlinear logit regression models were used to analyse differences between the intervention and control group in proportions of participants who displayed a clinically relevant change in the SF-36 scores from baseline to follow-up. Beneficial intervention effects on level of PA and overweight have previously been reported, regarding the study at hand [26, 27]. Additional regression models, where the PA and body weight variables were entered, stepwise, to test for possible influences on the hypothesised intervention effects on HRQoL, were therefore used. Cohen’s kappa was calculated as a measure of agreement between the two methods (‘change to top-score’ and ‘>20 point change’) for classifying individual increases in the SF-36 scores as clinically significant. This comparison was appropriate because the ‘change to top-score’ method is a modification of other novel methods, and the ‘>20 point change’ method is a more traditional method based on the standard error of the measurement. While the current study reports on secondary outcomes it was powered according to the primary aim. A priori sample size calculation showed that 70 individuals per group was sufficient to detect a group difference of 1.5 cm in change in waist circumference, with a statistical power of 80 % and alpha of 0.05.

### Ethical considerations

Ethical approval was obtained from Stockholm Regional Ethical Review Board (04-520/2) and all participants provided written informed consent prior to measurements [26, 27]. The trial is registered at ClinicalTrials.gov (NCT02320760).

## Results

### Baseline characteristics

The final study population consisted of 101 individuals who in 2006, aged 67 to 68 years, were randomised to the intervention (*n* = 47, 57 % women) or control (*n* = 54, 57 % women) group. There were no significant differences between the intervention and control group at baseline regarding anthropometrics, PA according to pedometry or self-report, sitting time and HRQoL, except for the SF-36 scale general health (diff = 10, *p* < 0.05, power = 0.62), in which the intervention group scored significantly lower than the control group (Tables 1 and 2). The frequency of participants scoring 88 or higher in the SF-36 scores at baseline did not differ significantly between the intervention and control group (Table 3). The flow of participants through the trial is depicted in Fig. 1 and baseline characteristics are described in Table 1.

**Table 1:** Baseline characteristics regarding physical activity, body weight, and sitting time

Variable	Intervention (*n* = 47)	Control (*n* = 54)
Sex (female/male (percent female))	27/20 (57 %)	31/23 (57 %)
Steps per day, pedometry	5390 (2791)	4980 (2763)
Body weight (kg)	88 (14.2)	88.3 (11.1)
Physical activity diary		
Sessions/week of at least moderate intensity^a^	2 (1–5)	2 (1–5)
Minutes/week of at least moderate intensity^a^	120 (0–220)	130 (40–215)
Questionnaire		
Sitting time (hours/day)	5 (3–7)	5 (4–7)
Leisure time physical activity		
Sedentary leisure time	13 %	11 %
Light activities ≥2 h/week	64 %	59 %
Regular moderate PA 1 to 2 · ≥30 min/week	15 %	20 %
Regular exercise ≥3 · ≥30 min/week	4 %	9 %
Follow-up data unavailable (n)	2	0

*Note*: All data expressed as mean (SD), median (Q1–Q3), number or percentage

^a^Defined as rate of perceived exertion (RPE) ≥12 [46–48]

**Table 2:** SF-36 baseline, follow-up and delta values and group differences

	Intervention			Control			
Variable	Baseline	Follow-up	Delta	Baseline	Follow-up	Delta	p
Physical functioning	85 (70–90)	88 (70–95)	0.0 (−8.3–10.0)	85 (70–94)	90 (80–95)	1.9 (−5.0–14.4)	0.58
Role physical	100 (75–100)	100 (100–100)	0.0 (0.0–25.0)	100 (75–100)	100 (75–100)	0.0 (−25.0–0.0)	0.15
Bodily pain	78 (75–100)	90 (58–100)	0.0 (−10.0–22.5)	78 (58–100)	79 (60–100)	0.0 (−12.5–20.0)	0.57
General health	70 (58–90)*	78 (65–85)	10.0 (−5.0–20.0)^†^	80 (65–85)*	80 (70–90)	0.0 (−10.0–15.0)	0.26
Vitality	63 (50–85)	75 (65–85)	5.0 (−10.0–30.0)	75 (65–85)	75 (65–90)	0.0 (−10.0–10.0)	0.10
Social functioning	100 (88–100)	100 (100–100)	0.0 (0.0–12.5)	100 (100–100)	100 (100–100)	0.0 (−0.0–0.0)	0.22
Role emotional	100 (67–100)	100 (100–100)	0.0 (0.0–33.3)	100 (100–100)	100 (100–100)	0.0 (−0.0–0.0)	0.08
Mental health	80 (64–92)	88 (76–96)	4.0 (−8.0–28.0)	90 (72–96)	88 (72–92)	0.0 (−8.0–8.0)	0.17
PCS	80 (67–88)	86 (76–90)	3.8 (−1.9–19.5)^†^	83 (73–90)	84 (69–92)	0.0 (−11.3–10.6)	0.12
MCS	84 (70–94)	90 (83–95)	4.4 (−2.4–23.3)^†^	89 (83–94)	89 (78–94)	0.0 (−4.0–4.9)	0.03

*Note*: Data expressed as median (Q1–Q3). P-values refer to the difference between intervention and control groups in delta SF-36, i.e. change from baseline to follow-up (Mann–Whitney U Test). PCS = physical component summary, MCS = mental component summary

*p < 0.05 regarding group difference at baseline (Mann–Whitney U Test)

^†^ *p* < 0.05 regarding change from baseline to follow-up within groups (Wilcoxon matched pairs test)

**Table 3:** Proportions per group, and odds ratios regarding clinically relevant improvements in SF-36 variables

Variable	Intervention (*n* = 47)		Control (*n* = 54)		Change to top-score^A^	>20 point improvement	
	SF-36 score <88 at BL (n (%))	Improved^A^(n (%))	SF-36 score <88 at BL (n (%))	Improved^A^(n (%))	OR (95 % CI)^B^	OR (95 % CI)^B^	Cohen’s kappa^C^
Physical functioning	28 (60 %)	10 (21 %)	36 (67 %)	16 (30 %)	0.64 (0.26–1.60)	1.17 (0.32–4.31)	0.79
Role physical	16 (34 %)	13 (28 %)	14 (26 %)	9 (17 %)	1.91 (0.73–5.00)	1.68 (0.67–4.30)	0.99
Bodily pain	26 (55 %)	11 (23.5 %)	31 (57 %)	12 (22 %)	1.07 (0.42–2.71)	1.34 (0.55–3.24)	0.87
General health	39 (83 %)	6 (13 %)	41 (76 %)	15 (28 %)	0.38 (0.13–1.08)	1.14 (0.42–3.11)	0.82
Vitality	39 (83 %)	7 (15 %)	42 (78 %)	8 (15 %)	1.01 (0.34–3.02)	4.32 (1.29–14.55)*	0.85
						5.03 (1.42–17.85)*^D^	
Social functioning	14 (30 %)	12 (25.5 %)	13 (24 %)	7 (13 %)	2.30 (0.82–6.45)	2.98 (0.72–12.24)	0.89
Role emotional	15 (32 %)	14 (30 %)	11 (20 %)	6 (11 %)	3.39 (1.18–9.74)*	2.85 (1.04–7.83)*	0.99
					4.25 (1.37–13.16)*^D^	3.46 (1.17–10.23)*^D^	
Mental health	26 (55 %)	10 (21 %)	25 (46 %)	10 (19 %)	1.19 (0.45–3.17)	2.30 (0.82–6.45)	0.86
PCS	38 (81 %)	10 (21 %)	34 (63 %)	9 (17 %)	1.35 (0.50–3.67)	1.70 (0.61–4.79)	0.83
MCS	29 (62 %)	18 (38 %)	25 (46 %)	11 (20 %)	2.43 (1.00–5.88)*	5.88 (1.54–22.42)**	0.83
					2.82 (1.09–7.33)*^D^	8.24 (2.03–33.49)**^D^	

*Note*: OR: Odds ratio; PCS: physical component summary; MCS: mental component summary

^A^Change to top-score: a baseline value <88 and follow-up value ≥88

^B^Nonlinear logit regression with ‘change to top-score’ or ‘>20 point improvement’ as dependent variable and group allocation as independent variable

^C^Agreement between the ‘change to top-score’ and ‘>20 point change’ classification methods in regard to the proportions of participants displaying clinically significant improvements in the SF-36 scores

^D^Change in body weight and self-reported leisure time physical activity as continuous covariates (only reported when these two covariates had a significant impact on the OR)

*p < 0.05

**p < 0.01

### Quality of life

None of the SF-36 scores met the assumptions of normality and all were left skewed, i.e. the scores were concentrated in the ≥88 “top-score” (see proportions in Table 3), thus non-parametric statistical methods were used. The intervention group improved significantly from baseline to follow-up in the scale general health (diff = 10, *p* = 0.03, power = 0.57), the PCS (diff = 3.8, *p* < 0.02, power = 0.35) and MCS (diff = 4.4, *p* = 0.02, power = 0.23). The control group displayed no significant changes from baseline to follow-up. The intervention group improved significantly more in the MCS (diff = 4.4, *p* = 0.03, power = 0.15) and displayed higher proportions of participants with relevant improvements in the role emotional scale (diff = 19 %, *p* < 0.02, power = 0.67) and MCS (diff = 18 %, *p* < 0.05, power = 0.52), in comparison to the control group.

The assumption of normality was not met for change in steps per day, body weight, PA of at least moderate intensity in sessions per week and in minutes per week, leisure time PA, as well as the changes in the SF-36 scores. We found change in body weight and change in self-reported leisure time PA to be the only delta variables fit for inclusion in the regression analysis. The delta PA and delta body weight variables were entered, stepwise, in the non-linear logit regression models to test for possible influences on clinically relevant changes in SF-36 scores. An increase in body weight and a decrease in leisure time PA had a statistically significant positive influence on the proportion of participants that exhibited a clinically relevant improvement in the SF-36 scores vitality, role emotional, and MCS.

## Discussion

The main finding from this study is that the Swedish PAP improved HRQoL, assessed by the SF-36 MCS, among older adults with abdominal obesity. These effects were, to some extent, mediated by changes in PA and body weight.

The group receiving PAP displayed beneficial changes in the SF-36 MCS and PCS scores from baseline to follow-up. The control group exhibited no such changes. The intervention group improved more than the control group in the scale Role emotional and the MCS, assessed as number of cases showing a relevant improvement. These findings are in line with studies on prescribed PA, including an uncontrolled study of the Swedish PAP [22]. In five Danish programs, exercise on prescription led to HRQoL improvements in 10 %–33 % of the participants [16], and in New Zealand prescribed PA has been shown to increase PA and HRQoL among inactive women [51].

Favourable intervention effects on the level of PA and overweight, regarding the study at hand, have previously been reported by Kallings and colleagues [26, 27]. Body weight, BMI, neck circumference and fat mass improved significantly in favour of the intervention group. In the current study body weight correlated strongly with, and gave almost identical results as, the other weight related measures. Body weight was therefore used as a proxy for these measures to minimise multiple testing. Somewhat surprisingly, the results in the study at hand show that decreases in self-reported leisure time PA, and increases in body weight, facilitated clinically relevant improvements in HRQoL. This is a conflicting finding because we hypothesised that an increase in PA and decrease in body weight would facilitate positive effects on HRQoL. However, there are several reasons to be cautious when interpreting these findings. First, the correlations between the delta PA, delta body weight, and delta SF-36 variables were mostly very weak and non-significant (not reported). Second, the Wald statistic (not reported) indicated that the contribution of each of these two predictors were small, and non-significant, and the regression models only improved if both of these covariates were included. Delta PA and delta body weight only had an effect in the already significant regression models. It could be that the present study was not powered to detect confounding effects. Further, pedometers, diaries and questionnaires are imprecise tools for measuring, foremost, intensity and duration of PA; therefore, we may have missed some actual changes in PA masking a stronger relationship with changes in HRQoL. Another possible explanation is the fact that some participants scored very high in some of the SF-36 scores at baseline, thus decreasing the likelihood of a clinically significant improvement, or improvements per se, at follow up.

There are several different determinants of HRQoL on an individual level, such as physical and mental health perceptions, functional status, social support [2], and intra- and interpersonal changes triggered by treatment and group support [52]. Such factors may be part of the mechanisms behind our results.

Despite trials such as the current one demonstrating that Swedish PAP has an effect on HRQoL, the true effect size may be underestimated. For instance, the placebo and Hawthorne effects could have an impact on the results reported here, masking a larger true effect [30]. On the contrary, practitioners in a regular clinical setting might not deliver Swedish PAP with the same confidence or emphasis as in this trial, which may lead to a smaller true effect. The latter seems less likely when comparing the present study to an uncontrolled study, conducted within the Swedish health care system [22]. That study presented significant increases in all but one of the SF-36 domains, while the present controlled study only identified effects on two of the SF-36 domains. Thus, the true effect of Swedish PAP on HRQoL may possibly be larger than reported here.

There are some limitations to this study. It was not statistically powered based on the self-report outcomes. HRQoL is often applied as a secondary outcome measure in exercise trials [39], and this study is no exception. Available sample size calculations based on normative Swedish data (power = 0.80, alpha = 0.05) show that the sample size in this study is sufficient to detect a 20-point between group difference at follow-up, and a 10-point difference between baseline and follow-up within groups, for all SF-36 scores, and a 10-point between group difference at follow-up for the scores physical functioning, general health, vitality, social functioning, and mental health [38]. The statistical power obtained regarding the results in the present study ranged from weak (0.15) to moderate (0.62). Low statistical power is thus a limitation in this study. A cut-off of 88 was chosen to determine a relevant change in the SF-36 and was inspired by the work of Velanovich [41], and Spratt [42]. However, as Spratt points out, the clinical importance of change is related to several factors, such as baseline score, age, gender, tobacco and alcohol use, medications, mood, and how well the patient can comply with the treatment. We consider the methods used in the study at hand conservative, and the SF-36 cut-off of 88 an appropriate indicator of a clinically significant change. The intention to treat approach had a more conservative impact on the results compared to simply excluding cases with missing data from analysis. Still, this may limit interpretation of the results. The SF-36 scale for general health differed significantly between the groups at baseline, which indicates a risk for regression to the mean. However, the delta values for general health did not differ significantly between the two groups, and we found no intervention effect on general health. Thus, regression to the mean does not seem to have affected the results.

Compared to Swedish normative data available for 65 to 69 year old men and women (*n* = 517) [38] our study sample had significantly higher mean scores for role physical (diff = 12, *p* < 0.001), and general health (diff = 5, *p* < 0.5), but did not differ for the other six SF-36 scores (PCS and MCS not included in the normative data base). Further, the participants in the present study were recruited from a larger randomly selected population sample. Thus, we consider our sample to be fairly representative of the Swedish population for this age group in regard to HRQoL.

Future studies evaluating the effect of PAP on HRQoL should be powered to detect clinically relevant changes and set HRQoL as a primary outcome. It is of importance to choose the most appropriate method, out of several available, for handling and statistically analysing SF-36 data. Objectively measured aspects of PA other than just steps per day, such as the use of accelerometers, may add quality.

## Conclusion

The Swedish Physical Activity on Prescription model may have a positive effect on mental aspects of HRQoL measured by the SF-36 among older men and women with abdominal obesity. The changes in HRQoL occurred independently of changes in PA, body weight, and sedentary behaviour. These findings support the clinical use of the Swedish PAP model.

## Abbreviations

BMI: Body mass index
CI: Confidence interval
ERS: Exercise referral scheme
IQR: Interquartile range
MCS: Mental component summary
OR: Odds ratio
PA: Physical activity
PAP: Physical activity on prescription
PCS: Physical component summary
HRQoL: Health related quality of life
Q1–Q3: Lower and upper quartile
RCT: Randomised controlled trial
RPE: Rate of perceived exertion
SD: Standard deviation
SF-36: The 36-item short form health survey
